# A Single-Scan, Rapid Whole-Brain Protocol for Quantitative Water Content Mapping With Neurobiological Implications

**DOI:** 10.3389/fneur.2019.01333

**Published:** 2019-12-20

**Authors:** Ana-Maria Oros-Peusquens, Ricardo Loução, Zaheer Abbas, Vincent Gras, Markus Zimmermann, N. J. Shah

**Affiliations:** ^1^Institute of Neurosciences and Medicine 4 (INM-4), Forschungszentrum Jülich, Jülich, Germany; ^2^Institute of Neurosciences and Medicine 11 (INM-11), JARA, Forschungszentrum Jülich, Jülich, Germany; ^3^JARA - BRAIN - Translational Medicine, Aachen, Germany; ^4^Department of Neurology, RWTH Aachen University, Aachen, Germany

**Keywords:** quantitative MRI, quantitative water content, quantitative macromolecular content, T_2_^*^, fast quantitative imaging, brain oedema, brain tumors, stroke

## Abstract

Water concentration is tightly regulated in the healthy human brain and changes only slightly with age and gender in healthy subjects. Consequently, changes in water content are important for the characterization of disease. MRI can be used to measure changes in brain water content, but as these changes are usually in the low percentage range, highly accurate and precise methods are required for detection. The method proposed here is based on a long-TR (10 s) multiple-echo gradient-echo measurement with an acquisition time of 7:21 min. Using such a long TR ensures that there is no T_1_ weighting, meaning that the image intensity at zero echo time is only proportional to the water content, the transmit field, and to the receive field. The receive and transmit corrections, which are increasingly large at higher field strengths and for highly segmented coil arrays, are multiplicative and can be approached heuristically using a bias field correction. The method was tested on 21 healthy volunteers at 3T field strength. Calibration using cerebral-spinal fluid values (~100% water content) resulted in mean values and standard deviations of the water content distribution in white matter and gray matter of 69.1% (1.7%) and 83.7% (1.2%), respectively. Measured distributions were coil-independent, as seen by using either a 12-channel receiver coil or a 32-channel receiver coil. In a test-retest investigation using 12 scans on one volunteer, the variation in the mean value of water content for different tissue types was ~0.3% and the mean voxel variability was ~1%. Robustness against reduced SNR was assessed by comparing results for 5 additional volunteers at 1.5T and 3T. Furthermore, water content distribution in gray matter is investigated and regional contrast reported for the first time. Clinical applicability is illustrated with data from one stroke patient and one brain tumor patient. It is anticipated that this fast, stable, easy-to-use, high-quality mapping method will facilitate routine quantitative MR imaging of water content.

## Introduction

Water concentration is highly regulated in the healthy human brain and changes only slightly with age and gender (1–3). The brain consists of roughly 80% water, distributed between different compartments. The normal adult human intracranial cavity (~1.4 L) comprises several compartments including blood (~100 mL), CSF (~100 mL) and brain parenchyma intracellular (~1.1 L), and interstitial (~100 mL) spaces (4). Water flows between these compartments in response to osmotic and hydrostatic forces.

In several diseases, the brain swells because excess water accumulates in the brain parenchyma. Cerebral oedema and brain swelling inevitably accompany ischemic infarcts and intracerebral hemorrhages and, when severe, may increase mortality to nearly 80% (5). Even in non-life-threatening stroke, the magnitude of brain swelling is strongly predictive of patients' functional outcome (6). Cerebral oedema and brain swelling occur in 20–30% of patients with acute liver failure and increase mortality to ~55% (7). Cerebral oedema and brain swelling after traumatic brain injury are estimated to account for up to 50% of patient mortality (8). Brain oedema, which can be life-threatening when very severe, is defined as a regional or global increase in water content. Understanding and treating brain oedema is a pressing clinical problem [for a recent review, see (9)]. Current treatments for brain oedema are inadequate and involve administering osmotic diuretics with transient benefit, corticosteroids, or performing invasive procedures such as decompressive craniectomy (10). These tend to be incomplete, non-specific, and short-lived measures (11). The reliance on non-specific treatments for brain oedema is a result of an incomplete understanding of specific cellular mechanisms by which brain water content is controlled under physiological conditions (12). However, recent advancements in understanding molecular mechanisms of oedema formation suggest that novel treatments may be close. There is mounting evidence that aquaporin-4 (AQP4) deficiency is associated with reduced cytotoxic brain oedema in mouse models, including water intoxication and focal cerebral ischaemia (13). In contrast to its beneficial role in cytotoxic oedema, AQP4 deficiency produces more brain swelling in mouse models of vasogenic oedema, including brain tumor, infusion of normal saline into brain extracellular space (ECS), and focal cortical freeze injury, the latter leading to a leaky blood-brain barrier (BBB) (14). AQP upregulation has also been detected in aggressive human tumors and it is believed that they might facilitate cancer spread (4). Aquaporin (AQP) inhibitors, in contrast, may slow tumor growth. These recent results and understanding (4) suggest possible applications of AQP-channel modulators—which are, however, yet to be developed—to be used for treating several brain conditions including trauma, tumor, hydrocephalus, and seizures. A non-invasive method for monitoring oedema in both research and clinical set-up would help to understand these mechanisms and implement treatment. Whereas, MRI in the clinic is not applicable as a means of constant monitoring, correlating MRI-based water content measurements at a few time points with indirect measures, such as thermal tissue conductivity (15), might provide means of introducing brain water content as an important parameter for monitoring patients with acute neurologic injury.

The gold standard for measuring water content of tissue is by invasive wet-dry measurements, where the difference (evaporated mass) is equated to water content, and the remaining solid mass largely to macromolecules. *In vivo*, in healthy tissue, the complement of water content in the voxel can thus be considered to largely reflect the macromolecular content of tissue (16). Changes in water content induced by disease can indicate either vasogenic or cytotoxic oedema, where extra-cellular and/or intra-cellular water content increases but the solid mass is not changing, or loss of solid mass (for example, by demyelination), where the percent water content in the voxel is increasing because its complement is decreasing. Consequently, a non-invasive measurement of water content *in vivo* is relevant to a multitude of neuroscientific questions which involve quantifying changes in the myelin content of the brain or, more generally, substance loss, for example investigating healthy aging and demyelinating diseases. With MRI, several pathologies/disorders such as alcoholism, haemodialysis, stroke, tumor, hepatic encephalopathy, and multiple sclerosis have been shown to lead to either local or global disturbances in water distribution and content (2, 17–23). Awareness of the correlation between water content measured by non-nuclear magnetic resonance (NMR) methods and the longitudinal NMR relaxation time, T_1_, in biological tissue has existed since the late 1970s (24–29) and has been used by several groups to derive surrogate water content values based on T_1_ (30–34). However, this is only an inferred quantity and changes in NMR relaxation times represent a composite of both water content and water structuring in the tissue under study (35). Despite the acknowledgment of this issue by several groups [see e.g., (36–39)], studies devoted to the development and use of accurate and quantitative water content measurement by MRI with whole-brain coverage have previously remained rare (2, 3, 40–43).

This is because, despite its apparent simplicity, several confounding factors, such as inhomogeneity of static, transmit, and receive fields, as well as ${\text{T}}_{2}^{*}$ decay, must be considered and corrected for when measuring water content with MRI. In recent years, interest in water content mapping has increased (16, 44–54). This is in line with a general growth in quantitative imaging with MRI and is also related to the fact that availability of higher-field systems with parallel imaging capabilities enable higher-SNR and faster data acquisition, respectively—both crucial for quantitative water content mapping. Nevertheless, at higher field strength several aspects related to the quantitation of water content, such as ${\text{T}}_{2}^{*}$ and ${\text{B}}_{1}^{+}$/${\text{B}}_{1}^{-}$ effects, become more significant and challenging to correct for than at lower fields (44–46). However, to date, the number of clinically relevant reports remains small (16, 22–24, 40, 45).

The simplicity of the signal equation and the relative transparency of its optimization partly account for the popularity of the variable flip angle (VFA) method for parameter mapping (16, 41, 43, 44, 52, 53, 55–61). In practice, as the VFA method is most frequently used with two angles it will be referred to as the two-point (2p) method in the following. We also employ the common notations ${\text{T}}_{2}^{*}$ = effective transverse relaxation time, α = flip angle, M_0_ = magnetization density, ${\text{B}}_{1}^{+}$ = transmit RF field, ${\text{B}}_{1}^{-}$ = receive RF sensitivity, SNR = signal-to-noise ratio, iPAT = acceleration factor for parallel imaging. Even after precision and accuracy optimization, the parameter space of the 2p method is vast. The different combinations of the relevant parameters TR/T_1_ and α can be classified into three different regimes: (a) TR/T_1_ < <1 used in conjunction with low flip angles; (b) an intermediate regime with TR/T_1_ ~1, one flip angle close to 90° and one lower one; and (c) TR/T_1_ >>1 (in practice, values of about 3 to 5) and flip angles close to 90°.

The TR/T_1_ < <1 regime for T_1_ values encountered *in vivo* is attainable with 3D mapping [regime (a)]. Care must be taken to avoid interference from unspoiled magnetization, which can have an immense effect on the steady-state signal (62, 63). In this case, TR values of several tens of milliseconds, i.e., comparable to the T_2_ value of white and gray matter (64), are beneficial for the quantitative powers of the 3D methods (52), but the acquisition times can become prohibitively long for *in vivo* acquisition unless parallel imaging is used. Despite a substantial reduction of the steady-state values due to TR/T_1_ < <1, the large number of encodings achieved in a 3D acquisition generates a high SNR in the images and maps (52, 58).

The intermediate regime TR/T_1_~1 is most suited for the 2p, 2D imaging [e.g., (43, 46)]. This regime benefits from the short acquisition times typical of 2D imaging and has sufficient SNR due to the relatively long TR values that allow high signal in the steady-state. However, in this case as well as for the 2p, 3D regime, knowledge of T_1_ is required to determine M_0_. Given that slice selection instead of slice encoding is performed, the SNR of 2p, 2D methods [regime (b)] and 2p, 3D methods [regime (a)] is comparable.

Whilst for TR/T_1_ < <1 or ~1 both scans are T_1_ and M_0_ weighted, this is not the case for the parameter range with TR/T_1_ >>1. For TR/T_1_ >>1, acquiring only one scan, instead of at least two, provides the information required for M_0_ mapping. The maximum possible steady-state value M_ss_ = M_0_ can be achieved in this regime. The number of corrections is minimized and the method can be considered the gold standard for proton density mapping (42). However, TR values of the order of 10 s or more can lead to prohibitively long measurement times if the in-plane resolution remains high. In this case, the use of parallel imaging is an absolute prerequisite. In our opinion, to date, this “gold standard” regime has not been exploited to its full potential.

In addition to the 2D or 3D acquisitions from which T_1_ and M_0_ can be calculated, which constitute the core of a VFA method in regimes (a) and (b), several additional scans are usually required. Indeed, corrections for transmit and receive inhomogeneity are involved in the calculation of water content maps (2, 3, 42, 43). Depending on the methods used, the measurement time for the corrections can exceed the acquisition time for the core data by a factor 2–3. In contrast, the method introduced here has the advantage that it does not require such additional scans to estimate the proton density parameter, M_0_.

We have developed a new method for water content mapping at 3T to make this important quantitative tool more accessible for clinical and neuro-scientific applications. Fast acquisition with a generally available sequence and robustness against SNR reductions were important criteria. Indeed, while the current standard clinical field is considered to be 3T and phased array coils allowing the use of parallel imaging are widespread, scanners operating at 1.5T, or even lower fields, are found in the clinic, and measurement time in patient studies continues to be an issue. Here, a conceptually simple, robust and fast single-scan method for water content mapping is presented and its potential is illustrated using various field strengths, scanners and coils, in both research and clinical environments.

## Materials and Methods

### Volunteers and Patients

Twenty-one volunteers divided into two cohorts were scanned on a 3T SIEMENS scanner. The first cohort included ten healthy male volunteers (mean ± std age 26.6 ± 1.2 years; range 24–28 years) scanned using a 12-channel receiver coil; the second cohort of eleven more volunteers (3 female; mean ± std age 29.8 ± 7.5 years; range 19–45 years) was scanned with a 32-channel coil. One additional volunteer (23 y.o.) was measured with the 12-channel coil on 3 different days for a total of 12 scans.

Five male volunteers (mean±std age 25.5 ± 3.9 years; range 20–29 years) were measured at two field strengths, 1.5T and 3T.

One brain tumor patient (male, 50 y.o.) was measured on a 3T hybrid MR-PET system after referral to our center for PET examination and pre-surgical MRI. Following surgery, histological examination of the tumor was performed.

One patient (male, 38 y.o.) participating in a prospective study was measured on a 3T PRISMA scanner 3 days after being admitted to the Aachen University hospital with symptoms of acute stroke and 4.5 days after supposed stroke onset.

All study protocols were approved by the review board of either the University Hospital Aachen or the University Hospital Cologne. Both patients and volunteers gave prior written informed consent to participate in the study, as per the requirements of the local ethics committee and conforming to the declaration of Helsinki (6th revision, 2008).

### Phantom

Phantom results were obtained on a so-called “revolver” phantom with 10 tubes, each containing a different, known mixture of H_2_O/D_2_O. The phantom was custom-made out of Perspex, and the tubes were standard 50 mL Eppendorf tubes. In experiments performed at the proton frequency, the D_2_O component is not detected and thus each tube has a different MR-visible water content. The T_1_ relaxation times were kept close to the *in vivo* values by addition of gadolinium-diethylenetriamine pentaacetic acid (Gd-DTPA) to each tube. The longitudinal relaxation times of the solutions in all tubes were determined in separate spectroscopic measurements to be below 3 s. No T_1_ saturation effects were thus considered. The revolver phantom loaded the RF coils similarly to a human head and a comparable B_1_ inhomogeneity was thus observed.

### MRI Protocol

All measurements were performed on scanners manufactured by Siemens Medical Systems GmbH, Erlangen, Germany. Measurements on the 21 healthy volunteers were performed on a 3T Tim-TRIO Siemens scanner equipped with gradients with a maximum strength of 40 mT/m per axis. A body coil was used for radiofrequency (RF) transmission and either a 12-element (10 volunteers and phantom) or a 32-element (11 volunteers) phased-array coil was employed for signal reception. The five volunteers from the field comparison study were scanned both in the 3T system with the 32-channel receiver coil and in a 1.5T Avanto system with body-coil RF excitation and signal reception using a 12-channel phased-array head coil.

The brain tumor patient was measured on a 3T hybrid MR-PET system consisting of a 3T Tim-TRIO scanner similar to the one described above, but with a Brain-PET insert and custom-built RF coil. A birdcage transmit and an 8-channel receive coil were used.

The stroke patient was measured on a 3T PRISMA scanner using a body-coil for transmit and a 20-channel head coil for signal reception.

In addition to the water content mapping scan, several sequences of clinical relevance were measured for both patients. Most importantly, a strongly T_2_-weighted sequence with fluid attenuation by inversion-recovery preparation (Fluid-attenuated inversion recovery, FLAIR) acquisition was used to identify the areas affected by tumor or stroke. Its acquisition parameters included: TR = 6,000 ms, resolution 1 x 1 x 2 mm^3^, TE = 403 ms, IR = 2,100 ms, α = 120°, parallel imaging (iPAT) factor 2, acquisition time TA = 5 min:20 s.

In all measurements, 3D shimming was performed with the manufacturer-supplied procedure used iteratively (4–8 times) until the full width half maximum (FWHM) of the signal from the whole head converged (in general to ~80 Hz). One 2D multiple-echo spoiled gradient echo (mGRE) scan was acquired with TR = 10 s and nominal flip angle of 90°. In the TRIO implementation, the manufacturer's sequence was modified to allow for the acquisition of 32 echoes. For the Avanto and PRISMA acquisitions, only 12 echoes were acquired with the default sequence. In all mGRE acquisitions, magnitude as well as phase images were saved. Other parameters were: field-of-view FOV = 200 x 162 mm^2^; slice thickness 1.5 mm, 1 mm gap; phase resolution 75%, matrix size = 192 x 117, in-plane resolution 1.04 x 1.38 mm^2^, interpolated online to 1.04 x 1.04 mm^2^; 57 slices; phase resolution 75%; 32 or 12 echoes; bandwidth BW = 280 Hz/px; TE_1_ = 3.87 ms; ΔTE = 4.08 ms. Parallel imaging was employed throughout, using an acceleration factor (iPAT) of 2. The acquisition time (TA) was 7:21 min.

The stability and reproducibility of the method were investigated by acquiring 12 separate scans from one volunteer using the TRIO scanner with the 12-channel coil with the protocol described above in 3 sessions over 1 week. Each scan involved complete repositioning and shimming.

The Tim-TRIO scanner and 12-channel coil were used for phantom measurements.

A 3D anatomical scan [three-dimensional magnetization-prepared rapid gradient-echo (MP-RAGE) sequence] with standard clinical parameters and 1 mm isotropic resolution was additionally acquired for the 5 volunteers measured in the field-comparison study. Its acquisition parameters included: TR = 2,250 ms, TE = 3.4 ms, TI = 1,100 ms, α = 15°, resolution 1 x 1 x 1 mm^3^, TA = 9 min:37 s.

### Concepts of Water Content Mapping

The general corrections required for accurate water content mapping have been discussed previously (2, 42–44, 46). The corrections relevant to the method presented here are summarized here.

#### Signal Equation

For the long TR values employed in the present study, T_1_ corrections for the steady-state of WM and GM are negligible. For T_1_ < 2 s and TR = 10 s (resulting in TR/T_1_ > 5), the steady-state correction is negligible for any flip angle. To describe such acquisitions, it is thus possible to use the signal model:

(1) $$\begin{array}{r} M=\;M_{0}\cdot \sin\alpha_{eff}\cdot e^{-TE/T_{2}^{*}}\cdot B_{1}^{-} \end{array}$$

with α_eff_ = α_nom_${\text{B}}_{1}^{+}$ where α_eff_ is the effective flip angle and α_nom_ is the nominal one, ${\text{B}}_{1}^{+}$ is a correction for deviations of the transmit field and ${\text{B}}_{1}^{-}$ is a correction for deviations of the receive field from unity. If the transmit and receive fields were homogeneous, ${\text{B}}_{1}^{+}$ and ${\text{B}}_{1}^{-}$ would be unity but these fields are generally inhomogeneous. ${\text{B}}_{1}^{+}$ also includes errors in the automatic calibration procedure of the scanner. In our implementation, α_eff_ is close to 90°, which maximizes SNR and has the additional advantage of minimizing the dependence of the measured intensity on ${\text{B}}_{1}^{+}$ inhomogeneity (*dM/d*α~0).

The main aim of our quantitative method is to relate M_0_ to percentage of tissue water content. In Neeb et al. (2), this is done at 1.5T by comparison with the signal from a reference probe of known water content. Therefore, the general form of the correction is (2, 43):

(2) $$\begin{array}{l} H_{2}O\text{​}\left[\%\right]=M\text{​}\left(\alpha_{eff}\right)\;\cdot \;f\text{​}\left(T_{2}^{*}\right)\;\cdot \;f\text{​}\left(B_{1}{}^{+};\alpha_{eff}\right)\;\cdot \;f\text{​}\left(B_{1}{}^{-}\right)\\ \;\cdot f\text{​}\left(reference;\alpha_{eff}\right) \end{array}$$

where *f(…)* are terms describing ${\text{T}}_{2}^{*}$, RF transmit, RF receive and probe calibration effects, addressed in detail in Neeb et al. (43).

We note at this point that the overall correction is factorized into multiplicative terms, each depending on one parameter only. The factor *f(reference;*α*)* is a scaling factor which describes the signal of pure water.

The correction terms in Equation (2), which depend on the RF field inhomogeneities, are expected to have smooth and long-range variations over the brain.

For the cerebral-spinal fluid (CSF), for which T_1_ saturation remains important due to the long T_1_ relaxation time of this medium, an additional steady-state correction factor needs to be employed and this is given by:

(3) $$\begin{array}{r} f\left(TR,\;T_{1},\;\alpha \right)=\frac{1-E_{1}}{1-\cos\alpha_{eff}\cdot E_{1}} \end{array}$$

with

$$\begin{array}{l} E_{1}=e^{\frac{-TR}{T_{1}}}. \end{array}$$

#### ${\text{T}}_{2}^{*}$ Correction

Two approaches were investigated. In the first approach (exponential model), the behavior of the signal intensity as a function of echo time was modeled with an exponential decay given by:

(4) $$\begin{array}{r} S\left(TE\right)=S_{0}\cdot e^{-TE/T_{2}^{*}} \end{array}$$

According to this model, the intensity of each echo can be corrected by the factor *exp(TE/*$T_{2}^{*}$) to give *S*_0_.

In the second approach (background gradients model), the inhomogeneities of the applied magnetic field were taken into account and partly corrected for as follows. The background gradients were calculated from the acquired phase images with an approach adopted from An and Lin (65), Dahnke and Schaeffter (66), and Bakker et al. (67). The gradients in each spatial direction, *i*, were calculated according to

(5) $$\begin{array}{r} g_{i}=\frac{1}{N}\overset{N}{\underset{n=1}{\sum }}\frac{\Delta \phi_{i,n}}{{\gamma \cdot \Delta x}_{i}{\cdot TE}_{n}} \end{array}$$

where Δϕ_i,n_ denotes the phase difference in the *i*-th direction (x, y, or z) at the *n*-th echo time TE_n_, Δx_i_ is the voxel dimension in *i*-th direction, and γ is the gyromagnetic ratio of the proton.

The data were corrected for the sinc modulation induced by the cross-slice dephasing (66) and modeled by:

(6) $$\begin{array}{r} {S\left(TE\right)}_{corr}=\frac{S_{0}}{sinc\left(\frac{\gamma }{2}\cdot g_{z}\cdot \Delta z\cdot TE\right)}e^{-TE/T_{2}^{*}} \end{array}$$

S_0_ and ${\text{T}}_{2}^{*}$ were then determined by exponential fitting of the corrected signal intensity.

For the calculation of the gradient maps in this work, only echoes with an intensity of at least half the intensity of the first echo were used.

Moreover, the maximum number of echoes included in the fit of the signal decay, from which M_0_ and ${\text{T}}_{2}^{*}$ were extracted, was determined for each voxel individually. Echo times were only included when dephasing produced by background field gradients (as given by γ · *g*_*z*_ · Δ*z* · *TE*) within the voxel did not exceed 0.8^*^π.

#### Normalization to Known Water Content

In our previous studies, water content in the brain was deduced by comparing the known water content of a reference probe after correcting for temperature differences. This becomes difficult at 3T and higher fields, mainly due to the effect of the B_1_ inhomogeneity correction. With the aim of minimizing the number of corrections and simplifying the method, the signal from CSF, considered to correspond to 100% water, was investigated as an internal standard. In this case, no temperature correction is required. However, when a flip angle of 90° is used, an additional steady-state correction must be included for CSF, since its T_1_ is comparable to that of free water. Based on the measured effective flip angle in CSF on the Tim-TRIO and a T_1_ value of ~4.3 s (68–70), a correction factor of 0.93 was calculated and used throughout. We mention that the automatic calibration (Siemens software) of the RF power on this scanner leads to a systematic reduction of the effective flip angle by a factor 0.8 of the nominal one. This factor is scanner dependent and needs to be determined, but remains constant for each scanner across volunteers.

#### Inhomogeneity Filtering Using SPM

Due to the multiplicative nature of the correction described in Equation (2), both M_0_ and the correction field can be directly estimated from the measured signal values using a probabilistic framework for segmentation (71). The unified segmentation combines image registration, tissue classification, and bias correction in a single generative model and optimizes its log-likelihood objective function. The model assumes that the brain image can be partitioned into WM, GM, CSF, and non-brain tissue classes whose original signal distributions can be described by a mixture of Gaussian distributions. Since the contrast in the measured long-TR images is predominantly proton density, we can rely on the observation (3) that the distribution of proton density in each class of brain tissue can be described by Gaussian functions.

The bias in signal amplitude, which is modeled in the unified segmentation, should be multiplicative and have a smooth spatial variation. It is evident from Equation (2) that the correction to the M_0_ map fulfills these requirements. The RF transmit field, ${\text{B}}_{1}^{+}$, varies smoothly across the head at this field strength, even when dielectric effects are considered (72). The influence of the receiver inhomogeneity of birdcage or phased array coils on brain images can be modeled and successfully corrected. This step has become a standard procedure in brain segmentation (71). In the generative model, the bias field is modeled by the exponential of a linear combination of cosine basis functions.

Bias field correction was performed using SPM12 (https://www.fil.ion.ucl.ac.uk/spm/).

Interestingly, this approach also corrected the field inhomogeneity from the phantom experiment, despite a lack of similarity between phantom and brain structure. The brain mask was generated using the brain tissue probability maps obtained from a first run of the SPM algorithm on the whole FOV: all voxels with a probability value higher than 0.25 in any kind of tissue (WM/GM/CSF) formed the brain mask. Tissue masks for each tissue type were calculated according to the following conditions:

(7) $$mask_{x}=\{\begin{array}{ll} 1, & P_{X}\geq 0.98\wedge P_{Y}<0.98\wedge P_{Z}<0.98\\ 0, & \;otherwise \end{array}$$

where X, Y, and Z represent the three tissue types WM, GM, and CSF in all possible permutations and P_X_ is the probability of tissue X.

#### Water Content Maps

All corrections described above were applied to the acquired data. The mean and standard deviation of the different quantities were calculated separately over all 10 volunteers scanned with the 12-channel coil and over the 11 volunteers scanned with the 32-channel coil. A steady-state correction factor of 0.93 was used at 3T TRIO and a factor 1.0 at 1.5T Avanto to describe the reduction in CSF intensity due to T_1_ effects and B_1_ distribution.

The magnetization density was derived from the long-TR scan after ${\text{T}}_{2}^{*}$ correction and subsequently corrected for RF inhomogeneity effects using SPM. Normalization to CSF was performed as follows: a histogram of the intensity of all voxels belonging to the CSF class with a probability of >98% according to the SPM segmentation was generated. The mode of the distribution of intensity values found in CSF was attributed to 100% water content (94% after correction for the T_1_ steady-state effect).

Additionally, for a single volunteer, 12 individual data sets were acquired and processed. All 12 volumes were coregistered with SPM12 and the average of the water content maps was calculated. For each voxel, the mean value and the standard deviation in the water content was calculated over the 12 scans.

For the stroke patient, the affected volume was manually delineated based on FLAIR and diffusion data, and the water content in the area was compared to that of the manually drawn corresponding region in the contralateral hemisphere.

### Data Analysis

Data processing and analysis were performed with in-house MATLAB scripts (2014a, Mathworks, Natick, MA, USA) and SPM12. Cortical parcellation of MP-RAGE data sets for 5 volunteers was performed using Freesurfer's *recon-all* pipeline [(73), http://surfer.nmr.mgh.harvard.edu/, version 6.0.0] to enable visualization of the water content distribution. The Destrieux atlas, which divides the cortical surface in sulci and gyri based on its curvature and cytoarchitecture (74), was used for the parcellation. Subsequently, MPRAGE and the quantitative maps were coregistered through means of an affine transformation, obtained using *bbregister*, a Freesurfer tool, with *flirt* initialization (75–77). The means and standard deviations of the quantitative maps of each of the 5 volunteers were calculated for each region in the parcellation atlas. The means were then projected on an average cortical surface provided by Freesurfer (fsaverage) to visualize changes in the quantitative maps between the different cortical regions.

### Statistical Analysis

The distributions of water content values obtained with the 12-channel coil and 32-channel coil were compared using balanced one-way ANOVA, as implemented in MATLAB. The distributions of water content obtained from the 5 volunteers scanned at both 1.5T and 3T were compared using a two-sample Kolmogorov-Smirnov test.

## Results

### Phantom Results

**Figure 1:**
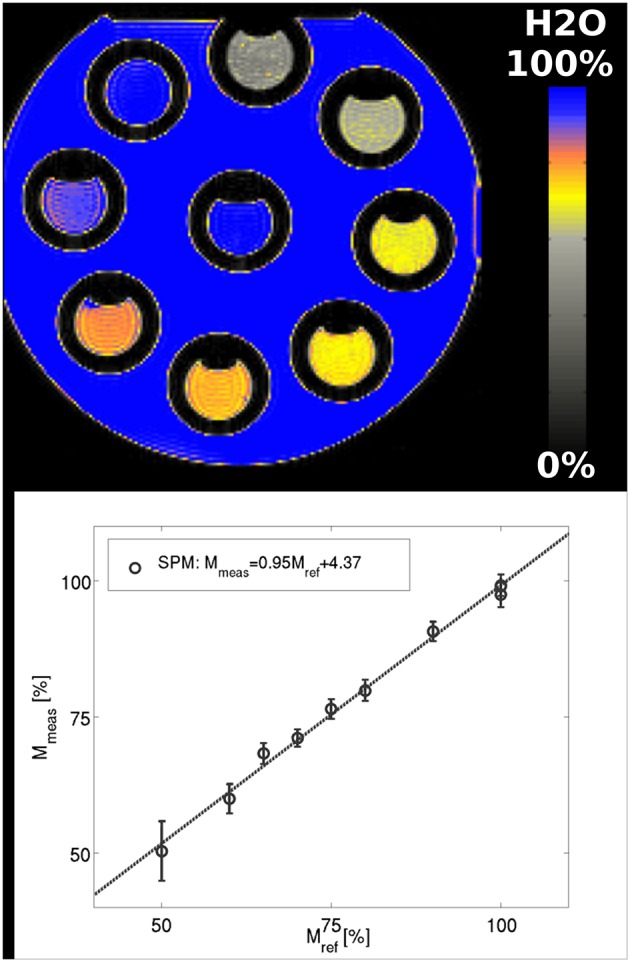
Phantom results using SPM-based filtering of the bias field. The function describing the linear relationship between the water content values obtained after ${\text{T}}_{2}^{*}$ correction and SPM filtering and the reference values is M_meas_[%] = 0.95*M_ref_[%]+4.37. The fit gives *r* = 0.997, *p* = 7.36^*^10^−9^.

A comparison of phantom results obtained with the current method and the known H_2_O values is shown in Figure 1. The function describing the linear relationship between the water values obtained after ${\text{T}}_{2}^{*}$ correction and SPM filtering is M_meas_[%] = 0.95^*^M_ref_[%]+4.37 (*r* = 0.997, *p* = 7.36^*^10^−9^). The agreement with the reference water values was within 1.5%. The mean value of the standard deviation within each tube amounted to 2.4%.

### *In vivo* Results: Healthy Volunteers Measured on 3T TRIO

#### ${\text{T}}_{2}^{*}$ Maps

**Figure 2:**
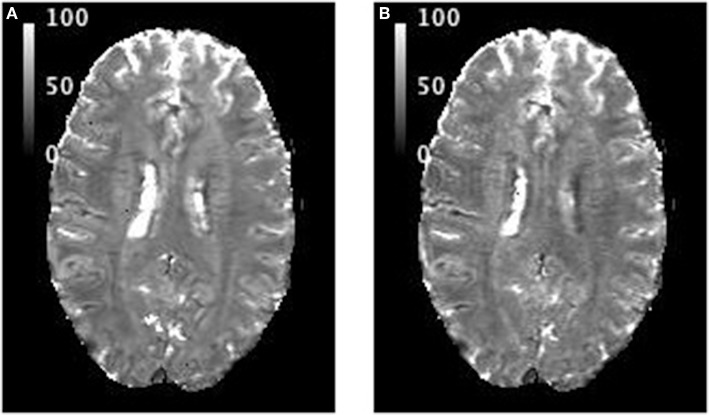
${\text{T}}_{2}^{*}$ maps showing the same slice from one volunteer: **(A)** after exponential fit to the original data; **(B)** when the effect of intra-voxel dephasing due to through-slice gradients was corrected first, followed by exponential fitting. All values are in ms.

${\text{T}}_{2}^{*}$ maps of a representative slice from one volunteer are presented in Figure 2A (exponential fit of original data) and Figure 2B (exponential fit following sinc correction). The mean ${\text{T}}_{2}^{*}$ value for the whole brain (tissue only), calculated from data on all 21 volunteers **(**10 from the 12-channel coil and 11 from 32-channel coil acquisitions), is ${\text{T}}_{2}^{*}$ = 52.4 ms and its standard deviation is 2.1 ms. The distribution can be reasonably well-described by a Gaussian fit with FWHM of 21 ± 1.5 ms for all volunteers. Using SPM-based segmentation of the brain, the mean and FWHM values over the 21 volunteers are: ${\text{T}}_{2}^{*}$(WM) = 50.0 ± 2.2 ms, FWHM(WM) = 13.2 ± 1.3 ms and ${\text{T}}_{2}^{*}$(GM) = 55.7 ± 2.6 ms, FWHM(GM) = 20.5 ± 1.4 ms.

#### H_2_O Maps

**Figure 3:**
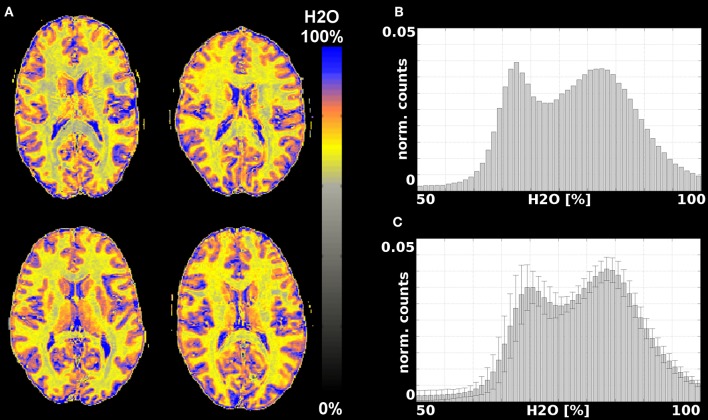
Results from measurements with a 12-channel coil. **(A)** Water content maps for a representative slice and 4 different volunteers obtained after ${\text{T}}_{2}^{*}$ and SPM correction; **(B)** histogram of the water content distribution in brain tissue for one volunteer; **(C)** average histogram for all 10 volunteers.

**Figure 4:**
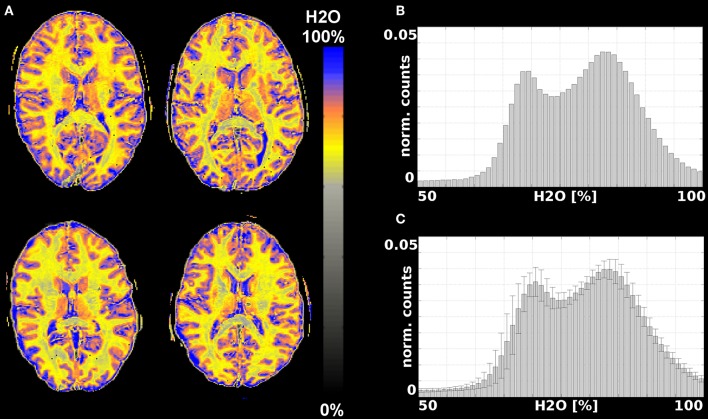
Results from measurements with a 32-channel coil. **(A)** Water content maps for a representative slice and 4 different volunteers obtained after ${\text{T}}_{2}^{*}$ and SPM correction; **(B)** histogram of the water content distribution in brain tissue for one volunteer; **(C)** average histogram for all 11 volunteers.

Representative water content maps are shown in Figures 3, 4. To illustrate the variability encountered in our population sample, images are shown for four volunteers. Figure 3 summarizes results from data acquired with the 12-channel coil. Selected water maps of anatomically similar slices for four volunteers are shown in Figure 3A, together with the histogram of the water content for one volunteer (Figure 3B) and the averaged histogram for the 10 volunteers (Figure 3C). Similar information for the 11 volunteers scanned with the 32-channel coil is presented in Figures 4A–C. The distributions shown in Figures 3C, 4C were compared using balanced one-way ANOVA, as implemented in MATLAB, and were found to be identical (*p* > 0.05). A two-sample Kolmogorov-Smirnov test was also applied to the cumulative distributions. The *p*-value for the Kolmogorov-Smirnov test was *p* = 1.00 for 64-bin histograms and *p* = 0.989 for 128-bin histograms. We consider this detail of binning sufficient to describe the water content distribution in healthy subjects.

Table 1 summarizes the global characteristics of brain water content obtained from our study. The values were calculated from data obtained for 10 volunteers measured with the 12-channel coil, for 11 volunteers measured with the 32-channel coil and from all data together (21 volunteers).

**Table 1:** Global characteristics of water content distribution in the white and gray matter of 21 volunteers.

	**WM mean mean ± SD**	**WM width mean ± SD**	**GM mean mean ± SD**	**GM width mean ± SD**	**mean(GM)/mean(WM)**
12-channel coil (*N* = 10)	68.9 ± 1.9	2.7 ± 0.4	83.7 ± 1.0	5.1 ± 0.5	1.21 ± 0.02
32-channel coil (*N* = 10)	69.3 ± 1.8	2.8 ± 0.4	83.6 ± 1.5	4.8 ± 0.2	1.21 ± 0.02
All volunteers	69.1 ± 1.7	2.8 ± 0.4	83.7 ± 1.2	4.9 ± 0.4	1.21 ± 0.02

*Ten volunteers were measured with a 12-channel coil, and the remaining 11 were measured with a 32-channel coil. All water content values are given in percent units*.

#### Test-Retest Stability

**Figure 5:**
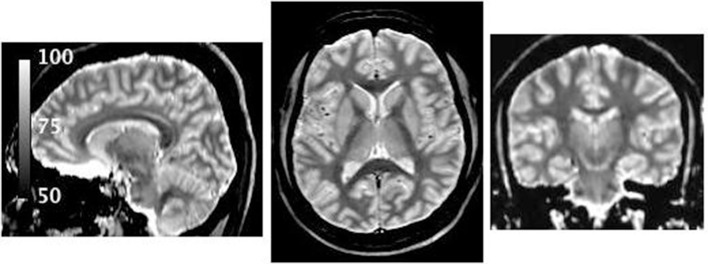
Water content map obtained after coregistration and averaging of 12 individual maps of the same volunteer. Due to the high SNR several anatomical features are visible, for example, the substructure of the thalamus and regions known to have high myelin content (7, 74).

**Figure 6:**
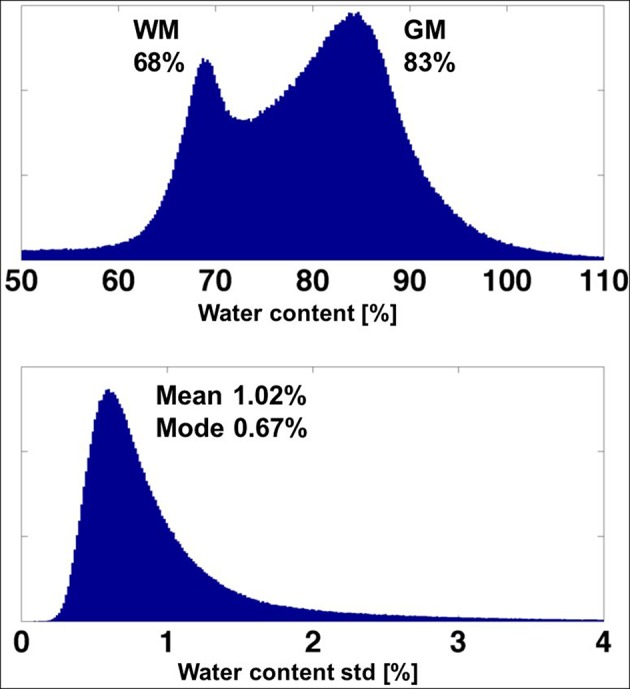
**(Top)**. Histogram of the water content obtained from the averaged (high SNR) map of one volunteer. **(Bottom)** Histogram of the voxel-based standard deviation of the water content over 12 measurements.

Figure 5 shows three orthogonal views of the water content map, obtained by averaging 12 scans. A histogram of the distribution of water content values and the SD of the water content in each voxel is shown in Figure 6.

The mean value and its SD over 12 scans of the white matter parameters were: mean 68.18 ± 0.33, FWHM of the distribution 3.14 ± 0.16. The corresponding numbers for the GM were: mean 83.32 ± 0.38, FWHM 5.17 ± 0.14. The mean value of the voxel-based standard deviation of water content (Figure 6) was found to be 1.02% and the most probable value (mode) was 0.61%.

### *In vivo* Results: Field Comparison

**Figure 7:**
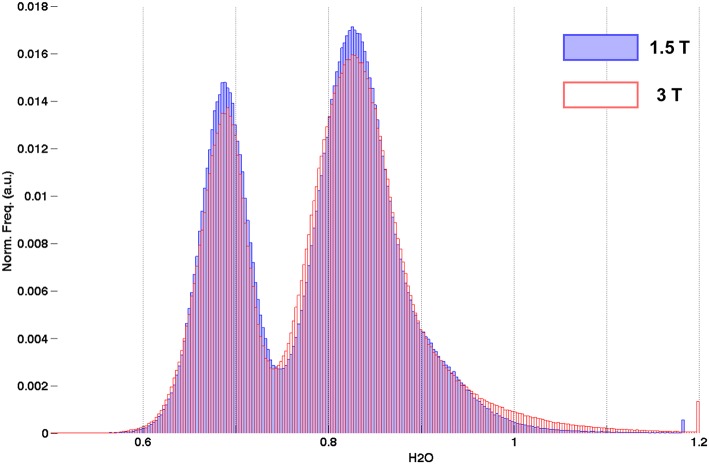
Cumulative histograms of water content from healthy volunteers measured at 1.5T (blue) and 3T (red).

Cumulative histograms of water content values are shown in Figure 7 for the 5 volunteers measured at two field strengths - 1.5 (red) and 3T (blue). Although the distributions appear very similar, they were found to be significantly different after performing a two-sample Kolmogorov-Smirnov test (*p*-value = 0).

The ${\text{B}}_{1}^{+}$ distributions at the two fields are shown in Supplementary Figure 1, displaying an increasing ${\text{B}}_{1}^{+}$ inhomogeneity with increasing field strength.

### *In vivo* Results: Distribution of Water Content and ${\text{R}}_{2}^{*}$ Values in Gray Matter

**Figure 8:**
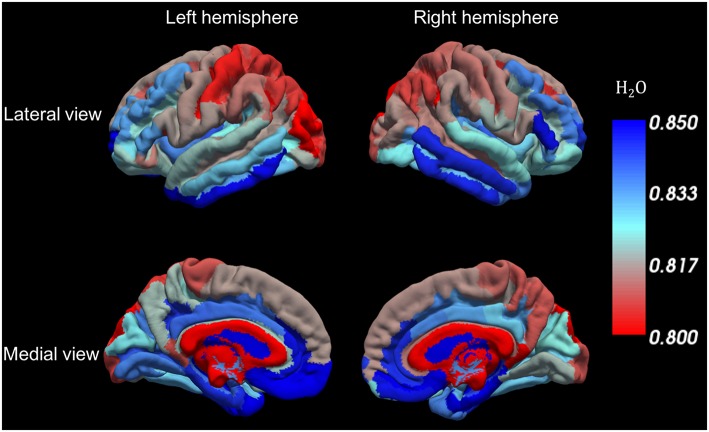
Water content variations along the cortical surface. Each color represents the average water content in each cortical ROI.

**Figure 9:**
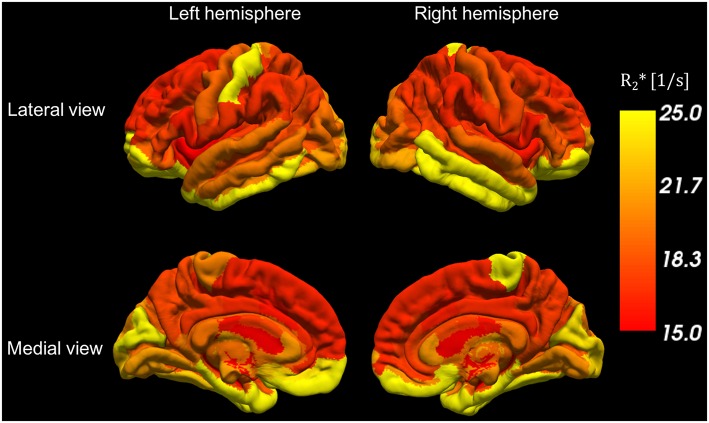
${\text{R}}_{2}^{*}$ variations along the cortical surface. Each color represents the average water content in each cortical ROI.

For each of the 5 volunteers with MP-RAGE scan data, parcellation based on Freesurfer was performed and the values from the quantitative maps were transferred to MP-RAGE space as described before. Visual inspection of the Freesurfer results was performed following the parcellation. No major errors were identified and therefore no manual editing of the Freesurfer parcellation was performed. The mean values were then projected onto the cortical surface of the Freesurfer template for each ROI, as shown in Figures 8, 9.

### *In vivo* Results: Measurements on Patients

**Figure 10:**
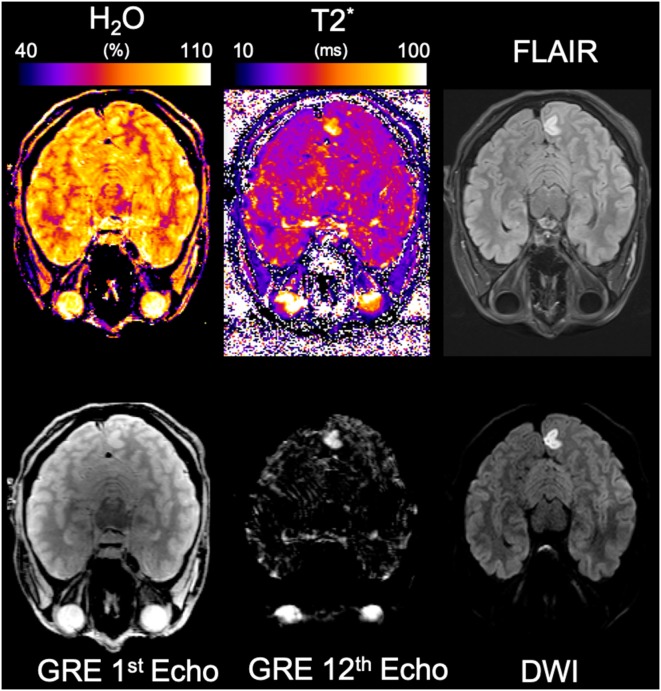
Results from the stroke patient. The same representative slice from the lesioned region is shown in each panel. The panels shown are, from left to right, top to bottom, water content, ${\text{T}}_{2}^{*}$, FLAIR, GRE 1st echo, GRE last echo, and diffusion-weighted imaging.

**Figure 11:**
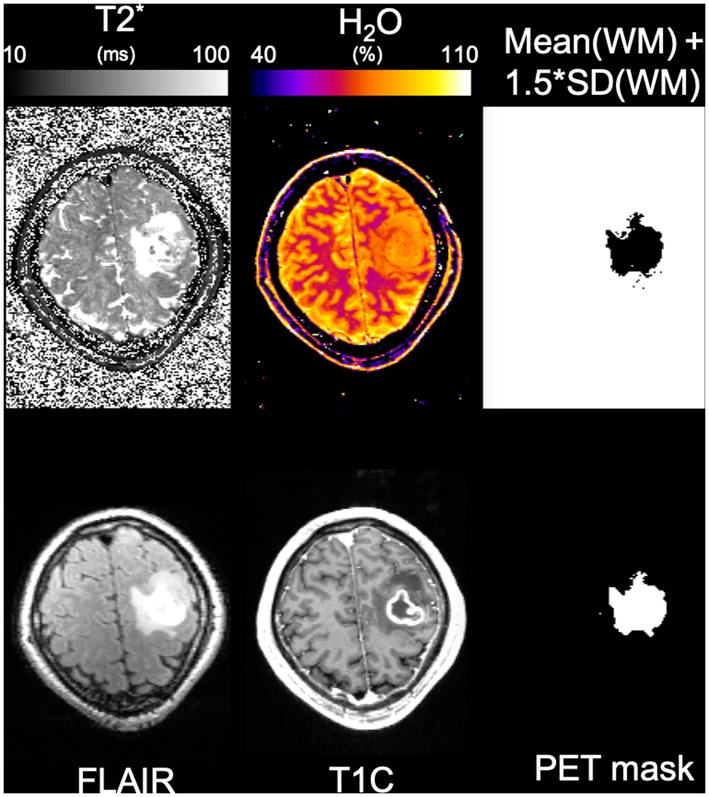
Results from the tumor patient. The same representative slice from the lesioned region is shown in each panel. The panels shown are, from left to right, top to bottom, T_2_*, water content, binary map obtained from the mean water content in WM plus standard deviation in WM, FLAIR, MP-RAGE post-contrast, and active brain mask obtained from PET.

The stroke patient was diagnosed with a 4-day old unilateral left occipital infarction. The histology result of the tumor patient revealed the mass to be a glioblastoma multiforme. Figures 10, 11 show water content and ${\text{T}}_{2}^{*}$ maps from representative slices through brain areas affected by stroke (Figure 10) or tumor (Figure 11). The quantitative maps are compared in each instance to the visualization of pathology provided by FLAIR (Figure 10) and MP-RAGE after contrast agent (Figure 11) and to the information provided by the original multi-echo data (Figure 10). Thus, the first echo is representative of the water content map, and the heavily ${\text{T}}_{2}^{*}$ weighted echoes (TE~${\text{T}}_{2}^{*}$ was selected) reflect a combination of T_2_ contrast similar to FLAIR and susceptibility anomalies (e.g., hemorrhages).

For the stroke patient, based on FLAIR and diffusion data, an affected volume of 3.41 mL was identified in the left occipital gray matter. Volume delineation was performed by an experienced neuroradiologist (O.N., who is gratefully acknowledged). The water content in this cortical area was measured as 89.5%, and in the anatomically corresponding contralateral area as 82.5%, showing an increase in water content of 7 percent units.

For the tumor patient, an active tumor volume of 16.65 mL was defined based on PET information as described in Oros-Peusquens et al. (78), and an additional volume of 18.80 mL affected by oedema was identified based on FLAIR.

The mean value of water content in the tumor was 80.3 ± 4.2% and 77.2 ± 4.1% in the oedema region. In contrast, the mean water content value for white matter in the contralateral hemisphere was mean(WM) ± SD(WM) = 69.8 ± 2.8%. Defining the “oedema” region as the region in which water content values exceed a threshold equal to mean(WM)+1.5^*^SD(WM) resulted in a masked area very similar to that based on FLAIR and FET-PET, as shown in Figure 10.

## Discussion

Benefitting from advances in scanner technology and image reconstruction, in conjunction with the increased SNR at a field strength of 3T compared to 1.5T, parallel imaging has made an otherwise largely conceptual “gold standard” for water content mapping clinically feasible. In addition to reducing the measurement time for a single scan with 1.04 x 1.38 mm^2^ in-plane resolution and TR = 10 s to below 10 min, the increase in field strength is known to enhance effects created by susceptibility variations and RF distribution, which in general need to be carefully characterized and corrected for. Here we have introduced a conceptually and experimentally simple water content mapping method that requires no additional measured corrections. Two postprocessing steps are required: an extrapolation to TE = 0 which corrects the effect of ${\text{T}}_{2}^{*}$ weighting and a heuristic, SPM-based intensity correction for the combined effects of ${\text{B}}_{1}^{+}$ and ${\text{B}}_{1}^{-}$. The effects of each are discussed below. Although similar approaches for the correction of either transmit (79) or receive inhomogeneity (80) have been used before, they have not been used to correct the combined effect of transmit and receive RF inhomogeneity together.

With our approach, all slow-varying inhomogeneities can be eliminated simultaneously and thus the need for time-consuming ${\text{B}}_{1}^{+}$ mapping is negated. The information required for ${\text{T}}_{2}^{*}$ correction is acquired at no additional time cost since TR = 10 s is long enough to allow for the acquisition of echo trains reaching to over a 100 ms. The usual limiting factor is the maximum number of echoes allowed by the sequence and/or the gradient duty cycle constraint. Furthermore, the ${\text{T}}_{2}^{*}$ information is intrinsically coregistered with the water content maps, since it is obtained from the same data set. For comparison, the measurement time reported by Volz et al. (44) for their water content mapping paradigm is 18 min per volunteer. In contrast, the time required to map water content and ${\text{T}}_{2}^{*}$ in the brain with our method is only 7:21 min and could be further reduced (see section Discussion). Preliminary results of this study were reported in Oros-Peusquens et al. (81).

### Parameter Optimization

The parameters were chosen to maximize SNR as the mapping of water content needs to be accurate and precise enough to be able to reflect variations in the few percent range. We base our discussion on Equations (1) and (3), which together reflect the signal equation for a spoiled gradient-echo sequence. We neglect the effect of ${\text{B}}_{1}^{+}$ and ${\text{B}}_{1}^{-}$ inhomogeneities. The maximum attainable signal in this sequence is M_0_, the equilibrium magnetization. This is achieved when T_1_ saturation effects are negligible and the flip angle amounts to 90°. For healthy volunteers scanned at 3T, all of the white matter, all of the deep gray matter and at least 97% of the cortical gray matter have T_1_ values below 2 s (64). Using a repetition time of TR = 10 s (5 times T_1_ = 2 s) the saturation effects on protons with T_1_ = 2 s are below 0.7%. We have deemed this situation acceptable and have chosen TR = 10 s and α = 90°. These parameters, in conjunction with the matrix size required for 1.04 x 1.38 mm^2^ in-plane resolution, imposed the use of parallel imaging with an acceleration factor of at least iPAT = 2 to keep the measurement time per scan below 10 min.

**Figure 12:**
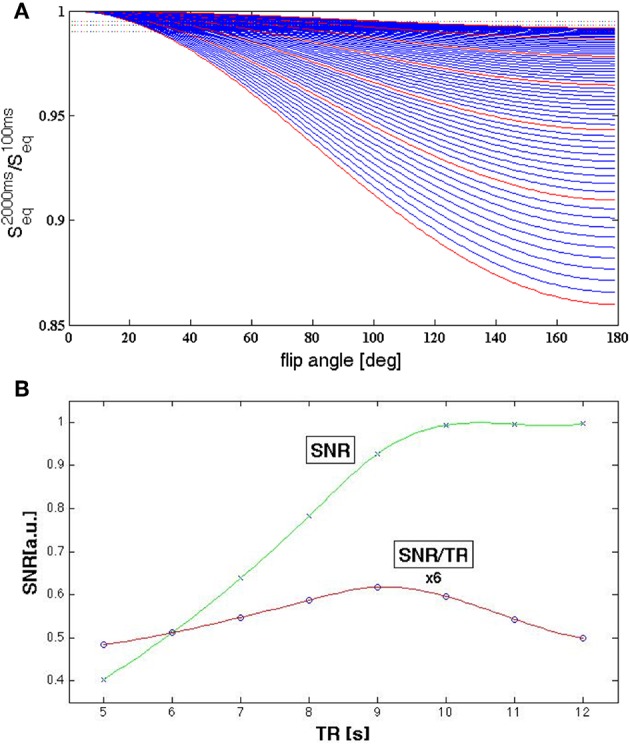
**(A)** Signal behavior for T_1_ = 2 s and different TR and α values. The red lines represent signal curves for integer values TR = 5, 6,…, 10, 11 s and the dotted lines correspond to saturation values of 1, 0.7, and 0.5%. A saturation level of 1% is reached for α = 90° at TR = 9.2 s; a saturation level of 0.5% for TR = 10.5 s. **(B)** SNR per unit time (circles, red line is drawn to guide the eye) for acquisitions with (TR, α_max_) determined such that the saturation effect remains below 0.7% for T_1_ = 2 s. The optimum is between 9 and 10 s. SNR (crosses, green line to guide the eye) increases strongly up to TR = 10 s after which it changes little with TR.

This (TR,α) combination is also very close to providing the optimum SNR per unit time for a single-scan water content mapping acquisition. We calculate SNR / unit time under the constraint that T_1_ saturation for all included (TR, α) combinations should remain below 0.3% for T_1_ = 2 s. The signal dependence on α for different TR values and T_1_ = 2 s is shown in Figure 12A. This constraint allows the calculation of the maximum flip angle, α, at which the saturation is kept below the mentioned limit for different TR values and also ensures maximal SNR. The optimum SNR / TR, shown in Figure 12B, is reached for values between TR = 9 s and TR = 10 s, but TR = 10 s has a higher SNR. However, for water content mapping, SNR/unit time does not have the same impact as SNR alone. If the method is not precise enough to detect changes in water content of the order of 1%, measurement speed alone is not useful.

### ${\text{T}}_{2}^{*}$ Correction and Maps

The extrapolation of the measured signal to zero echo time, the ${\text{T}}_{2}^{*}$ correction, is normally based on an exponential fit to the measured signal decay. However, it has been shown that a polynomial extrapolation is more appropriate in cases where the effect of through-slice gradients on the signal is large (2, 3).

Alternatively, an exponential pulse can be employed for slice selection (82) and leads to an exponential signal decay.

In our implementation, a manufacturer-provided sinc pulse was used for slice selection. The sinc modulation created by through-slice gradients can be calculated based on field information provided by multi-echo phase images and corrected (82). The decay of the corrected signal can then be described as exponential.

Reasonably thin slices (1.5 mm) were used in the present study and careful shimming was performed on each volunteer before the start of the gradient echo measurements. In the vast majority of the brain voxels, the through-slice gradient correction has effects well below 0.5% [Figure 7 of (43) and own results at 3T, data not shown]. The mean water content values over the whole-brain tissue were found to vary by less than 3% following correction for background field gradients using the sinc factor. For regions affected by strong B0 field inhomogeneity, such as the sinuses and ear canals, this correction is however important and leads to changes of up to 10%.

We can conclude that, in most regions, a simple exponential fitting will suffice. We have found, however, that the voxel-based determination of the number of echoes included in the fit has an effect on the overall water content values via the CSF calibration. Since this determination is based on phase data and additional processing, described before, we recommend including a large number of echoes in the fit of the slowly-decaying signal from CSF to improve the fit accuracy for water content mapping studies where calibration is performed on CSF. For tissue ${\text{T}}_{2}^{*}$ fitting, a reduced subset of echoes (roughly up to ${\text{T}}_{2}^{*}$) should be considered. These simple modifications should allow accurate water content values to be obtained in most brain regions with CSF-based calibration using a simple exponential fit and magnitude-only data.

Despite being slightly longer due to the thinner slices used in the present study, the mean ${\text{T}}_{2}^{*}$ values for different tissue classes (see section Results) are in good agreement with 3T values previously reported (64). In pathologies, ${\text{T}}_{2}^{*}$ can be used as a surrogate T_2_ measure to identify regions of increased T_2_ (for example oedema, stroke).

### SPM-Based Bias Correction

As already noted, one advantage of the long-TR water content mapping method presented here is that all corrections shown in Equation (2) are multiplicative and involve independent variables. Therefore, both M_0_ and the correction field can be directly estimated from the measured signal values using the probabilistic framework for segmentation provided by SPM12 (71). Furthermore, each term in the series of multiplicative corrections can be calculated from measured data or estimated by SPM. We have investigated the feasibility of correcting the effects of both ${\text{B}}_{1}^{+}$(BC) and ${\text{B}}_{1}^{-}$(PA) fields using SPM12.

### Calibration Using the CSF Signal

While the position of the mean value of the water content distribution is not crucial for characterization of localized variations in the water content (e.g., oedema surrounding a tumor, MS lesions), it is extremely relevant for clinical applications involving generalized low-grade oedema (40). In this case, the comparison between the absolute value of the GM and WM water content for healthy volunteers and patients becomes important. Furthermore, the stability of the calibration is a crucial factor in measuring very small variations and increases the sensitivity of the method to pathology. External standards were successfully used for calibration at 1.5T (40). However, in the present method, the inhomogeneity correction is based on intensity values measured in the brain. The bias field outside the brain, where calibration probes are typically placed, would have to rely on extrapolated values. We have investigated this possibility in several studies and found the method to lead to unacceptably large variability in water content values between volunteers. Furthermore, placement of standards in the FOV and/or attached to the head of the volunteer adds to the complexity and duration of the measurement. These factors were avoided by using the CSF signal as a reference and assuming that the water content of CSF is identical to that of pure water and that the T_1_ relaxation time of CSF was subject independent. A closer look into the properties of CSF is, therefore, required.

Although normal CSF largely consists of water, our previous studies (2, 3, 43) have failed to identify a narrow water peak corresponding to CSF, whereas the distributions for WM and GM were quite well-separated. The same holds for T_1_ and ${\text{T}}_{2}^{*}$ values. This is probably because the relaxation properties of CSF are very different from those of tissue, and it is difficult to optimize the mapping parameters and/or the sensitivity of the method to deliver equally accurate results for tissue and CSF. Here we relied on the SPM-based segmentation to define the properties of CSF. The mode of the CSF signal distribution was determined. Although the distribution is not Gaussian, but very broad and asymmetric when all voxels from the CSF mask are included, the properties of voxels from the lateral ventricles are very similar and give rise to a well-defined peak, which is approximately Gaussian and which we characterize by its maximum (the mode of the entire CSF distribution). The value of the mode was defined to correspond to 100% water content. Since the nominal flip angle in our measurement was 90°, chosen such that SNR in brain tissue is maximized and B_1_ dependence is minimized, a slight T_1_ saturation effect is expected for CSF. It results in a correction factor of 0.93 using T_1_ values from the literature (69, 70) and a factor of 0.8 between the effective and the nominal flip angle, caused by the Siemens automatic calibration procedures on the TRIO scanner.

The field-independent T_1_ value for CSF of ~4.3 s, reported by Hopkins et al. (68), and by Rooney et al. (69), support the interpretation that CSF consists of approximately 100% water. A large effect of proteins on its T_1_ values would most likely give rise to a field dependence. Substantial changes in protein content in a patient population cannot, however, be excluded, and should be carefully investigated.

### Water Content Maps

#### Phantom Results

The mapping method was investigated using phantom results. A good correlation between the measured and known water content of the tubes was found, as shown in Figure 1. This demonstrates that the bias field correction does not affect contrast; i.e., it is not removed from small and intermediate-sized structures. On the short distance scale, the measured water content correctly shows changes from 100% to as little as 20% over the few mm separating the inside of a tube from the main container. On an intermediate structure scale, the values inside each tube are constant within ~2% and the contrast between tubes is maintained, as demonstrated by the good agreement between the MRI-measured and known water content values. However, the smoothly varying bias field can be successfully removed while maintaining the homogeneity of the water values inside the main container.

#### *In-vivo* Values

We address in the following various aspects influencing the quantitative power of the method. Whenever possible, we compare the proposed method with results from the literature, obtained either with alternative MR-based methods, or with invasive techniques. Good agreement with published values was found (see below), whereas the shorter measurement time, the stability and robustness against noise are clear advantages of the present method.

#### Accuracy

The water content measurements for WM and GM presented here are in very good agreement with values given in the literature. Based on a correlation analysis between T_1_ and water content, Fatouros et al. reported a water content value for posterior white matter of 69.6% and a GM water content in the head of the caudate nucleus of 80.3% (34). Results from invasive water content measurements in biopsy samples range from 68.7 to 71.6% for WM and from 80.5 to 84.6% for GM (31, 34, 83, 84). Other sources estimated WM water content to be around 68–71% and that of GM to 80–85% [see Table 4.2 of (85); Table 1 of (46)]. *In vivo* studies in humans (46, 54, 80) estimated the mean water fraction of WM to be around 69–70% and that of GM at 83%. These values are all in very good agreement with the average water content of 69.3 ± 1.4% for WM and 83.8 ± 0.8% for GM, as determined here. Some remnant contamination by CSF of voxels including cortical gray matter might have a slight effect on the GM mean value.

The centroid values are also consistent with previous values obtained at 1.5T (2, 3, 43). The small differences could be due to the fact that segmentation of brain tissue into white and gray matter was previously (2, 3, 43) performed using a simple cut-off in T_1_ values, and such a procedure has difficulties with assigning the basal ganglia to the GM class. In the present study, segmentation is based on the more sophisticated procedure included in SPM12 (71).

Water content has been shown to depend on age and gender (2). Therefore, same-sex volunteers of similar age were chosen for the study with the 12-channel coil, in order to avoid variability of water content in addition to the inaccuracies of the method.

#### Precision

The water content maps obtained after SPM-based bias field corrections show a very consistent picture. Normalization based on CSF water content was used to calculate all values listed in Table 1 and delivered highly consistent results. In order to assess the performance of the SPM-based bias field correction, we investigated the variability in the water content maps when different field distributions need to be reproduced.

The bias field obtained from the SPM segmentation is included in the Supplementary Material and shown in Supplementary Figure 2a for 4 volunteers measured with the 12-channel coil and in Supplementary Figure 2b for 4 volunteers measured with the 32-channel coil. Since the same body coil was used for RF transmit, the differences in the correction largely reflect the changes in the receive field. Despite the substantial differences in the receive inhomogeneity distribution of the 12- and 32-channel coils, the histograms of water content values, averaged over 10 and 11 volunteers, respectively, are very similar (Figures 3C, 4C). One-way ANOVA comparison of the mean values shows that the distributions do not differ significantly (*p* < 0.05). The same result is obtained when applying a two-sample Kolmogorov-Smirnov test to check whether the two distributions are different (*p*~1.0).

Although we have only tested two coil configurations out of the many possibilities, the constancy of the water content results for these substantially different receiver arrays inspires confidence in the bias field correction.

As seen from Table 1, using data from all 21 volunteers, the mean values of water content in WM and GM, the widths of the distributions and the ratio of the means all have very small standard deviations of below 2%. We can conclude that the physiological variation of water content within the population studied here, with a narrow age range, is very small. This is in accordance with previous results obtained at 1.5T with a different method (3).

The fact that the mean values of the water content in WM and GM, which depend on the reference value chosen for calibration, have a variability similar to that of the ratio of the two values, which is calibration-independent, inspires confidence in the CSF-based calibration.

#### Stability

The test-retest stability of the current method was found to be excellent. The voxel-based SD of water content over the whole brain and 12 independent measurements shows a most probable value of 0.6% and a mean value of 1% (Figure 6). The variations in the mean water content of WM and GM were 0.3–0.4%. These values are substantially smaller than the variability for all 21 volunteers (1.2–1.7%) and demonstrate that the method should be able to detect variations of the order of 1% in water content in longitudinal studies.

The histogram of the voxel-based SD in water content, shown in Figure 6, also points to the presence of regions with higher variability (SD of 2–4%). Not surprisingly, these values correspond to the regions affected by strong B_0_ inhomogeneities. The main reason for this variation is the inadequacy of the exponential fit, whether performed on original data or after sinc correction, which includes only first-order field inhomogeneities. However, it is possible that higher-order field inhomogeneities may also contribute to the variation and a different, non-exponential, extrapolation to TE = 0 might be necessary even after sinc correction. We stress the fact that the number of voxels affected by a sizeable variability is very small, as demonstrated in Figure 5, where the regions close to large susceptibility deviations show slightly elevated (and erroneous) water content values. For most practical purposes, however, this effect can be neglected.

#### SNR Effects

The aim of comparing water content distributions on the same volunteers scanned at two different field strengths was 2-fold. On the one hand, it offers a way to investigate the influence of SNR on the accuracy of the water content maps using 3T as a reference. The exact influence of SNR on the accuracy of the water content estimation is difficult to determine by purely theoretical considerations or signal simulations, since an important part of the method is the bias field correction by SPM, making it difficult to estimate error propagation and sensitivity to noise. The proposed field strength (1.5 vs. 3T) comparison is an experimental way of studying the influence of SNR on water content values. The fact that a 12-element receiver coil was used at 1.5T, whereas a 32-channel coil was used at 3T, further enhances the SNR disparity. Additional effects arise as a result of the different performances of the coils for parallel imaging due to both the different number of elements and also the field dependence of the g-factor (86).

A typical SNR value, defined as the mode of signal distribution divided by mode of noise distribution, in the first echo at 1.5T was 52, whereas at 3T it was 97. The effect of the lower SNR in the original contrasts on the maps is the broadening of the distributions. However, even at 1.5T, the two characteristic WM and GM distributions remain distinct. This is a clear advantage of the present method, which utilizes the whole available magnetization [up to a sqrt(2) x g-factor reduction due to the necessity to use parallel imaging]. The water content results are thus less sensitive to noise than those from e.g., 2-p methods based on a substantially reduced steady-state signal and more elaborate fitting.

The second aim of the comparison was to assess the performance of the method at 1.5T in terms of clinical applications, as a large number of 1.5T scanners are still available world-wide in clinical environments. The introduction of this simple, robust and short quantitative protocol in clinical routine would help to study brain oedema and a plethora of neurological afflictions. We would like to point out that the method is potentially applicable to other body parts or other objects, but the inhomogeneity filtering by SPM would require replacement with a more general method, e.g., N4ITK inhomogeneity correction (87).

#### Distribution in White and Gray Matter

Due to its high SNR, one very interesting and visible aspect in the average water content map (Figure 5) is that low water content values characterize the presence of highly-myelinated fibers in white matter, for example in the corpus callosum. These are the typical regions for which a high myelin water fraction has been reported (88).

The most probable cause for the low water content in these regions (around 4 percent units below white matter average) is that the high myelin water fraction also implies high macromolecular (myelin non-water) content (89). On average, less water and higher macromolecular content characterize these voxels. We cannot exclude, however, a possible influence from faster relaxation of myelin water. This issue, as well as the correlation of water content with myelin water fraction and the characteristics of fibers described e.g., by diffusion MRI data, requires further investigation.

Aspects investigating changes of water content in fiber tracts, as defined by diffusion, have been reported by Mezer et al. (16).

Very good contrast between white and gray matter is visible in the water content maps and has been recognized by virtually all the previous studies of brain water content. Although the exact reason is not clear, better geometrical packing of fibers in white matter and higher macromolecular content associated with myelinated axons should account for some of these 14 percent unit differences.

A novel aspect of water content distribution becomes apparent in Figure 8, which shows average water content values in cortical ROIs identified by Freesurfer. Here we only present data from the 5 volunteers for which anatomical scans were performed, and the details of the distribution might change somewhat with the inclusion of a large number of volunteers. Contrast in water content between neighboring cortical regions is easily recognized (e.g., in the frontal and occipital lobes). A table of the mean values in the different ROIs is included as (Supplementary Table 1). Supplementary Figure 3 is a representation similar to Figure 8 depicting the coefficient of variation of the water content in each ROI.

Measuring water content of the brain, defined as the fraction of voxel volume occupied by water, also provides a measure of its complement, the remaining content of the voxel. The protons which are not being detected by imaging systems (fast relaxing species) are tightly bound in macromolecules, together with most of the other abundant elements in the brain (e.g., C, P, N), which cannot be detected at the proton resonant frequency. The contribution of mobile ions—such as Na, K, Ca, Cl, at millimole/L concentrations—and trace elements to the voxel composition is much smaller and will be neglected. The macromolecular content of tissue can thus be estimated from water content without any need for further modeling (16, 69). It is interesting to point out that this is not the case for the bound proton fraction derived with quantitative magnetization transfer (qMT), where simplifications are needed (90–94). For example, the properties of different types of macromolecules need to be averaged, resulting in an “effective bound pool.” Water content thus appears to be a better measure of total macromolecular content of tissue, whereas the bound proton fraction (derived from qMT) reflects those molecules which have an effect on water properties (equilibrium magnetization, relaxation times).

Whereas, qMT can account for other parameters of interest, such as magnetization exchange rate and relaxation times of the bound proton pools, the bound proton fraction is by far the parameter with demonstrated clinical relevance and is also most likely relevant to brain parcellation based on cytoarchitecture.

The contrast seen in Figure 8 thus also depicts the different concentration of macromolecules in different cortical regions, of which perhaps the most interesting are constituents of myelin. It is known from myelin staining of histological sections (95–97) that cortical gray matter also includes myelinated axons. Myelin content of gray matter shown by stained sections is lower but comparable to that of white matter. However, T_2_-based myelin water measurements with MRI (88) usually fail to identify the typical short-T_2_ “myelin water” component in cortical gray matter which is commonly observed in white matter. Using the present method, we can easily assess variations in water content within the white matter, which appear to be associated with variations of myelin content [see Figure 5 and Supplementary Table 1, and also reported by Mezer et al. (16)]. It is, therefore, a working hypothesis that water content distribution in cortical gray matter might reflect myelination of different cortical regions.

We would like to highlight the reduced water content in the highly myelinated somatosensory cortex and high contrast to the immediately adjacent motor cortex in Figure 8. The water content in white matter regions, such as corpus callosum, also shows region-dependent variability and an interesting low-high-low pattern from the genu to the splenium along the corpus callosum (Supplementary Table 1).

We note that the distribution of water content in the human cortex described here is similar to that of another index of myelination, magnetization transfer ration (MTR), recently reported by Hunt et al. (98). However, discussion of these several intriguing aspects is outside the scope of the present manuscript. At this stage, the visualization and tentative discussion are aimed at highlighting the potential of the method.

#### Correlation With ${\text{R}}_{2}^{*}$

The distribution of ${\text{R}}_{2}^{*}$ values in the cortex is visualized in Figure 9 and shows region-specific values and contrast between cortical regions. The ROI-based coefficient of variation of ${\text{R}}_{2}^{*}$ is shown in Supplementary Figure 4. In order to assess whether the information follows that of water content, the correlation between ${\text{R}}_{2}^{*}$ and 1-H_2_O was investigated for all cortical regions (Supplementary Figure 4). If all the data points are included in the analysis, the Pearson correlation coefficient was found to be relatively low, *r* = −0.21. Indeed, ${\text{R}}_{2}^{*}$ is expected to be affected by other factors besides water content. Intra-voxel dephasing caused by field inhomogeneities are one important source, causing identical water magnetization distributions to show different signal decays for different field distributions over the voxel. Another important influence on ${\text{R}}_{2}^{*}$ is the pattern of distributions of transverse relaxation sources within the voxel.

#### Clinical Aspects

Clearly, brain oedema, defined as a regional or global increase in water content, is the most natural application for water content mapping in a clinical environment.

Cerebral oedema occurs in a variety of clinical emergency conditions, including blunt head trauma, episodes of ischemia and hypoxia and Reye's syndrome (99, 100). It is also present in metabolic disorders such as diabetes or hepatic encephalopathy (101).

Persistent, severe brain oedema may complicate treatment of other medical problems and be life-threatening. Current therapies for brain oedema include diuretic, hyperosmotic, and steroidal modalities (10). These tend to be incomplete, non-specific, and short-lived measures (11). The reliance on non-specific treatments for brain oedema is a result of an incomplete understanding of specific cellular mechanisms by which brain water content is controlled under physiologic conditions (12), and a non-invasive method for monitoring oedema in both research and clinical set-up would help to understand these mechanisms.

Even at the most pragmatic level, the method can benefit e.g., oncological applications by allowing for the quantitative delineation of regions affected by oedema, as shown in Figure 11. This is easily possible based on the very accurate characterization of the values measured in healthy volunteers and by defining a threshold of variation above which the regions can be considered oedematous.

Evolution of oedema with time after stroke onset, which has been investigated to some extent in animal models (102), is still poorly studied in clinical work on humans.

In our measurement on a subacute stroke patient (4.5 days after stroke onset) we show that increases in water content in the stroke area average 7% and are easily measurable. The first and last echo of the multi-echo acquisition are very suitable for use in a clinical environment, offering a rough estimate of water content and T_2_/${\text{T}}_{2}^{*}$ increases without any need for post-processing. We would like to point out that fingerprinting methods (103), which are considered disruptive technology in clinical research, do not have this advantage. Indeed, all the contrasts on which quantitative maps are based are not anatomically meaningful images in current fingerprinting techniques with similar acquisition times (103).

### Perspectives

#### Measurement Time Reduction

To ensure widespread clinical applicability of this quantitative method, it would be desirable to reduce the acquisition time even further. This is achievable using the standard mGRE sequence. For patient groups where movement is a major problem, if highly segmented RF arrays are available, acquisitions with higher acceleration for parallel imaging can be performed, for example with iPAT = 4. This would approximately preserve the total measurement time, whereas the measurement time per scan would be halved. Alternatively, shorter TR values in conjunction with lower flip angles could be used (78). The acquisition time for a single scan, in this case, drops to around 4 min, reducing the likelihood of head movement. The necessary SNR can be achieved by averaging two acquisitions, if necessary, after coregistration (104). Whole-brain coverage, which allows for 3D coregistration, is a plus in this case.

More efficient k-space sampling strategies could also be used to reduce measurement time. For example, an EPI-type read-out could be employed, although we would recommend using low EPI factors (e.g., a factor of 3) to preserve the accuracy of the ${\text{T}}_{2}^{*}$ information. This would result in a very substantial speed-up (e.g., factor of 3), which can be used in addition to parallel imaging with sequence modifications. Further, ultra-fast acquisitions with model-based reconstruction can be applied to water content mapping (105, 106), although clinical implementation of such advanced methods might be slow. Of course, the precision and accuracy of water content mapping will need to be reassessed after every substantial change of protocol.

#### Applicability at Higher Fields

Although the combined transmit/receive inhomogeneity was found to be quite substantial even at 3T, we anticipate that the method will remain usable at even higher fields—for example, 4, 4.7, and 7T. At ultra-high fields (9.4T and above), where the dielectric resonance effects become very pronounced (107), it is likely that B_1_ shimming / tailored excitation and an adequate receiver coil combination would become mandatory before application of bias field correction to filter out the remaining inhomogeneity. Whether the existing algorithms can be used for these purposes or whether a different description of the remaining inhomogeneity is required remains to be assessed.

#### Further Quantities

The long TR of the method allows for the acquisition of a long echo train, even when a large number of slices for full brain coverage is acquired. A multi-component analysis of the echo train is feasible, similar to that reported before using gradient-echo data (108, 109). The fact that we additionally map the absolute water content allows calculation of not only the myelin water *fraction* (MWF) but also the myelin water *content*. This information is very valuable in cases such as multiple sclerosis where both demyelination (lower MWF) and inflammation (higher water content) contribute.

By combining parameters obtainable from the data acquired with this method (110) and performing necessary corrections (111), a better characterization of the microstructure of tissue can be achieved in a multiparametric space. Indeed, tissue ${\text{R}}_{2}^{*}$ values, magnetic susceptibility and electric conductivity (reflecting free ion content) can be derived from this single-scan data set and correlated with water content values (112).

#### Applications

Given the current near absence of clinically used quantitative methods, we believe that this fast and easy to use method will contribute to our understanding of brain properties and of psychiatric and neurological diseases substantially. We propose that the method could be used for clinical and neurological applications where changes in the macromolecular proton fraction are of interest. Although quantitative magnetization transfer is showing promise in clinical applications (93, 94), water content mapping as offered by this method is faster, with direct biological significance and is much less affected by model hypotheses and simplifications than qMT.

Novel aspects are revealed by the distribution of water content and R2^*^ values in the cortex (Figures 8, 9). Detailed studies need to be performed to confirm or refute the correlation of cortical water content with myelin content since other factors such as cell density and cell content can contribute.

Should it prove to be correct, it opens entirely new possibilities of assessing the effects of neurodegeneration and demyelination in the human cortex based on changes in its water content.

## Limitations

Limitations specific to some particular issues have been discussed above but will be briefly reiterated and elaborated upon.

The main assumptions of the method are:

1. Measured ${\text{B}}_{1}^{+}$ and ${\text{B}}_{1}^{-}$ inhomogeneity corrections can be replaced by SPM bias field filtering;
2. The CSF region used for calibration (mainly consisting of lateral ventricles) has homogeneous T_1_ and B_1_ properties in all volunteers;
3. The echo train decay can be described by a mono-exponential decay;
4. The exponential decay can be refined with a sinc correction to account for substantial b_0_ inhomogeneities.

While these assumptions have eliminated the need for additional calibration measurements, and result in a method which is fast, conceptually simple and easy to implement, they are a potential source of errors.

1. It remains to be seen whether SPM can indeed correct for any transmit/receive coil configurations. an additional concern is that smooth water content variations in the brain, potentially generated by disease, might be erroneously described as bias field and removed.
2. The use of CSF as 100% water calibration standard could prove to be problematic in patient studies if the properties of CSF, more specifically its T_1_ relaxation, change with disease. For CSF calibration, we have used a field-independent T_1_ value of 4.3 s throughout.
3. The existence of water pools in brain tissue with distinct T_2_/${\text{T}}_{2}^{*}$ properties has been long known (88). This results in a deviation from mono-exponential signal decay with echo time. Whereas, the effect of myelin water is small (10% or less of the total signal, and contributing only to early echoes below 15 ms), water pools with different properties than those of healthy tissue might be present in pathologies (89).
4. The effects of the B0 field inhomogeneities on the signal evolution are not confined to that of the through-slice inhomogeneity. in addition, the latter might not be sufficiently described by a first-order approximation as constant gradient (66, 111).

Some additional concerns relate to use of the proposed method at high and ultra-high field. The assumptions regarding ${\text{B}}_{1}^{+}$ and ${\text{B}}_{1}^{-}$ will still hold at UHF, but the distributions will become more complex and it remains to be seen how corrections proposed here will work. Moreover, T_1_ increases at higher field and, therefore, adhering to the constraint of TR = 5T_1_ will increase the measurement time, although this could be ameliorated by using higher parallel imaging accelerations (iPAT factors).

Quite generally, partial volume effects will necessarily reduce the accuracy of the method at tissue boundaries; higher resolution imaging could be an answer but at the expense of increased acquisition times.

## Conclusions

This study demonstrates the possibility of performing accurate and precise water content mapping with high **(**1.04 × 1.38 × 1.5 mm^3^) spatial resolution of the whole brain using only one scan with a multiple-echo gradient echo sequence. The data processing steps are simple and involve easily obtainable software. The acquisition time for one scan, with the parameters used here, is ~7 min, making the method easily applicable to patients where the influence of disease on the properties of CSF must be investigated beforehand.

Clinically relevant changes in water content are often of just a few percent units, making stringent constraints on the required accuracy and precision of quantifying methods.

The accuracy of the present method has been tested on phantom measurements and *in vivo* measurements on 21 volunteers at 3T. The precision of the method is reflected in the small variability of mean values per tissue class obtained in a healthy population (2%), which probably mainly reflects physiological variability.

The stability of the method was found to be excellent, with variations in the mean values of ~0.3% and mean voxel-based SD of 1%; these values are based on the comparison of 12 scans for a single volunteer. Thus, the method should be able to characterize changes of the order of 1% in longitudinal studies well.

An important part of the method is the correction of the combined transmit and receive bias fields, which is done entirely by post-processing. The influence of the receiver coil on the quantitative water content distribution was found to be negligible. Here we compared a 12-channel and a 32-channel coil, considered to be representative of the current and the near-future clinical standard. This finding gives confidence in the robustness of the SPM12 bias-field correction and the capacity of the algorithm to describe the inhomogeneity features of modern receiver arrays.

When a highly sensitive receiver array is available, the measurement time can be reduced even further by using higher acceleration factors for parallel imaging.

The robustness of the results against lowering SNR was assessed by comparison of 1.5 and 3T data on 5 volunteers. The mean values of water content do not differ significantly between fields, even though the distributions are not identical, suggesting that the discrimination power of the method when comparing healthy and diseased populations is already attained at 1.5T.

Water content values in cortical gray matter are depicted for 5 volunteers and show a specific area-related distribution, suggesting unexplored potential for the study of neurological afflictions related to demyelination and/or cell loss.

The clinical applicability of the proposed mapping method is illustrated with data sets obtained from one stroke patient in a fully clinical environment and one brain tumor patient.

It is anticipated that this easy to use water mapping method will aid widespread quantitative MR imaging of water content. The short measurement time, which could be further reduced depending on the application, should facilitate the use of quantitative water mapping even in critical applications such as pre-surgical tumor investigations and acute or post-acute stroke.

## Data Availability Statement

The datasets generated for this study will not be made public. Processed data, which contain potentially sensitive information, are available upon request due to ethical restrictions, approved by the Ethics Committee of the RWTH Aachen University, to the Forschungszentrum Jülich. Consent was only gained to share the acquired data with the head of study and the responsible supervisory authority. Data and results presented in this manuscript will be available upon request via Prof. Dr. Günther Schmalzing, Ethics Committee of the Medical Faculty, RWTH Aachen.

## Ethics Statement

This study was carried out in accordance with the recommendation of the University Hospital Aachen and University Hospital Cologne ethics committees with written informed consent from all subject. All subjects gave written informed consent in accordance with the Declaration of Helsinki. The protocol was approved by the review board of both the University Hospital Aachen and the University Hospital Cologne.

## Author Contributions

A-MO-P contributed with the development of the method, data acquisition, data processing and manuscript writing. ZA contributed with additional data acquisition. MZ and VG contributed with data processing. RL contributed with data processing, results visualization, and manuscript editing. RL, ZA, VG, MZ, and NS contributed with revision of the manuscript.

### Conflict of Interest

The authors declare that the research was conducted in the absence of any commercial or financial relationships that could be construed as a potential conflict of interest.
