# Supplementary material for: A Single-Scan, Rapid Whole-Brain Protocol for Quantitative Water Content Mapping With Neurobiological Implications
Source: Front Neurol. 2019 Dec 20;10:1333. doi: 10.3389/fneur.2019.01333 (PMC6934004; doi:10.3389/fneur.2019.01333)
Supplement: Supplementary file 2 [file Presentation_1.PPTX]

## Slide 1
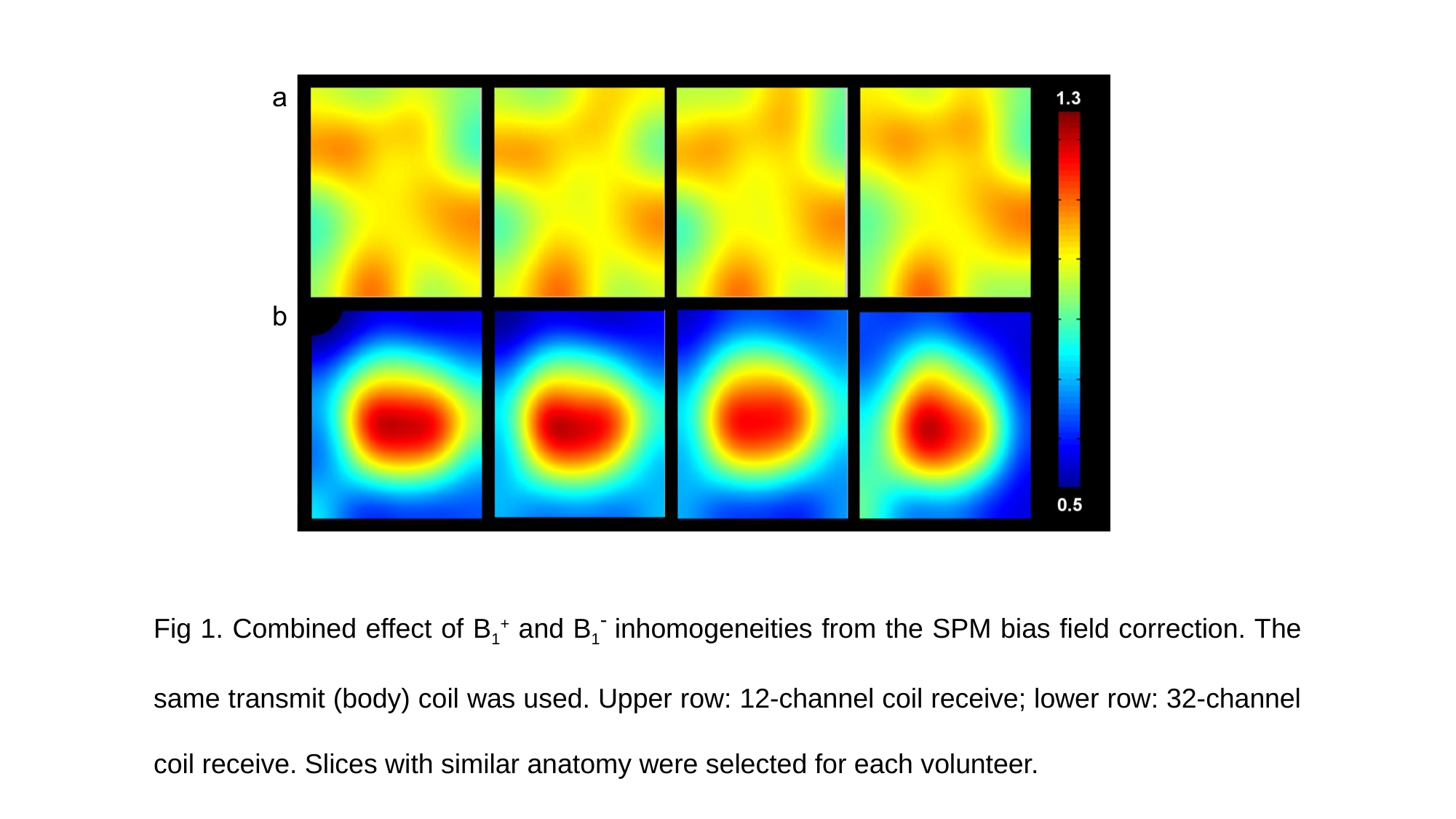

Fig 1. Combined effect of B1+ and B1- inhomogeneities from the SPM bias field correction. The same transmit (body) coil was used. Upper row: 12-channel coil receive; lower row: 32-channel coil receive. Slices with similar anatomy were selected for each volunteer.

## Slide 2
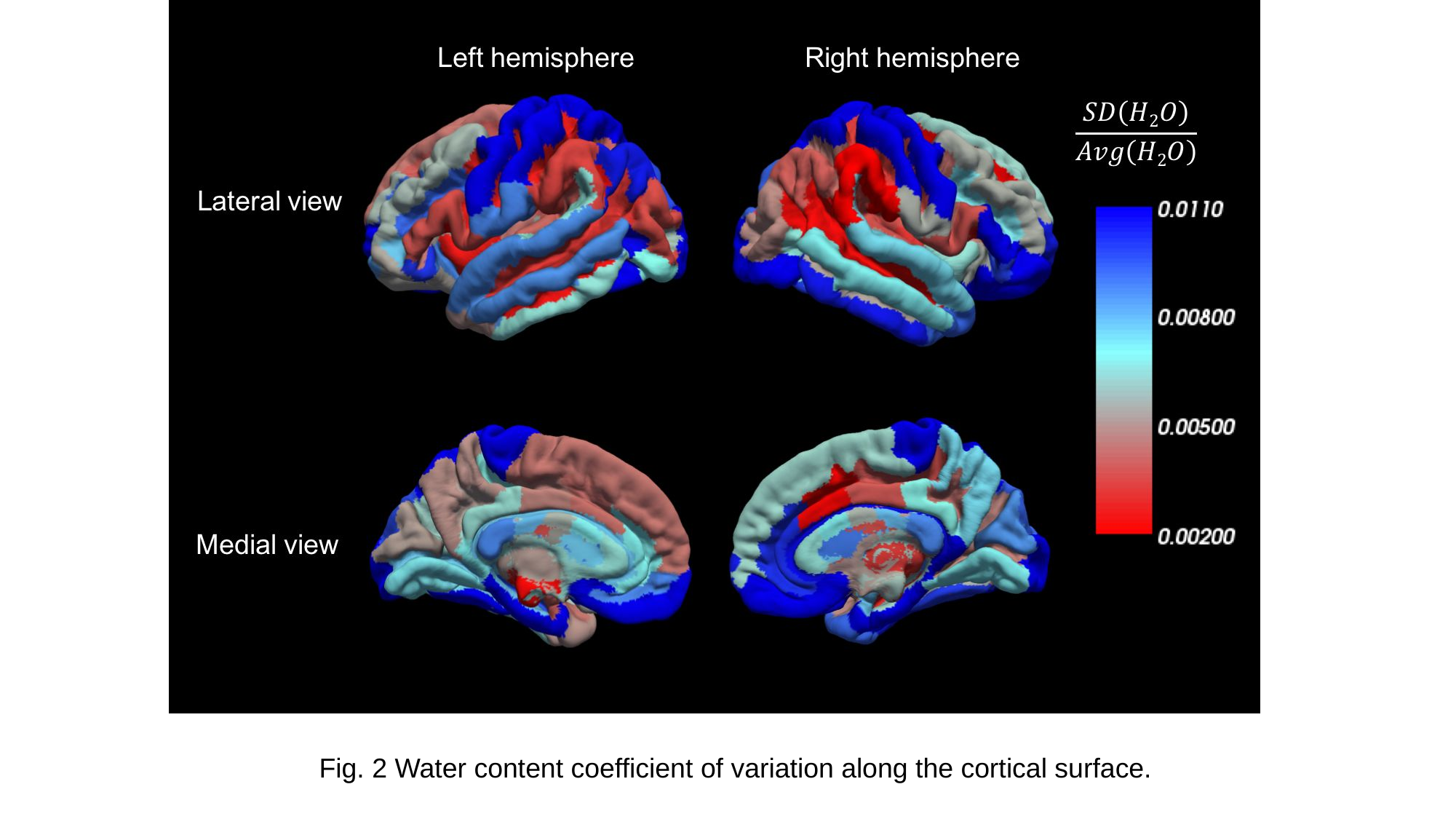

Fig. 2 Water content coefficient of variation along the cortical surface.

## Slide 3
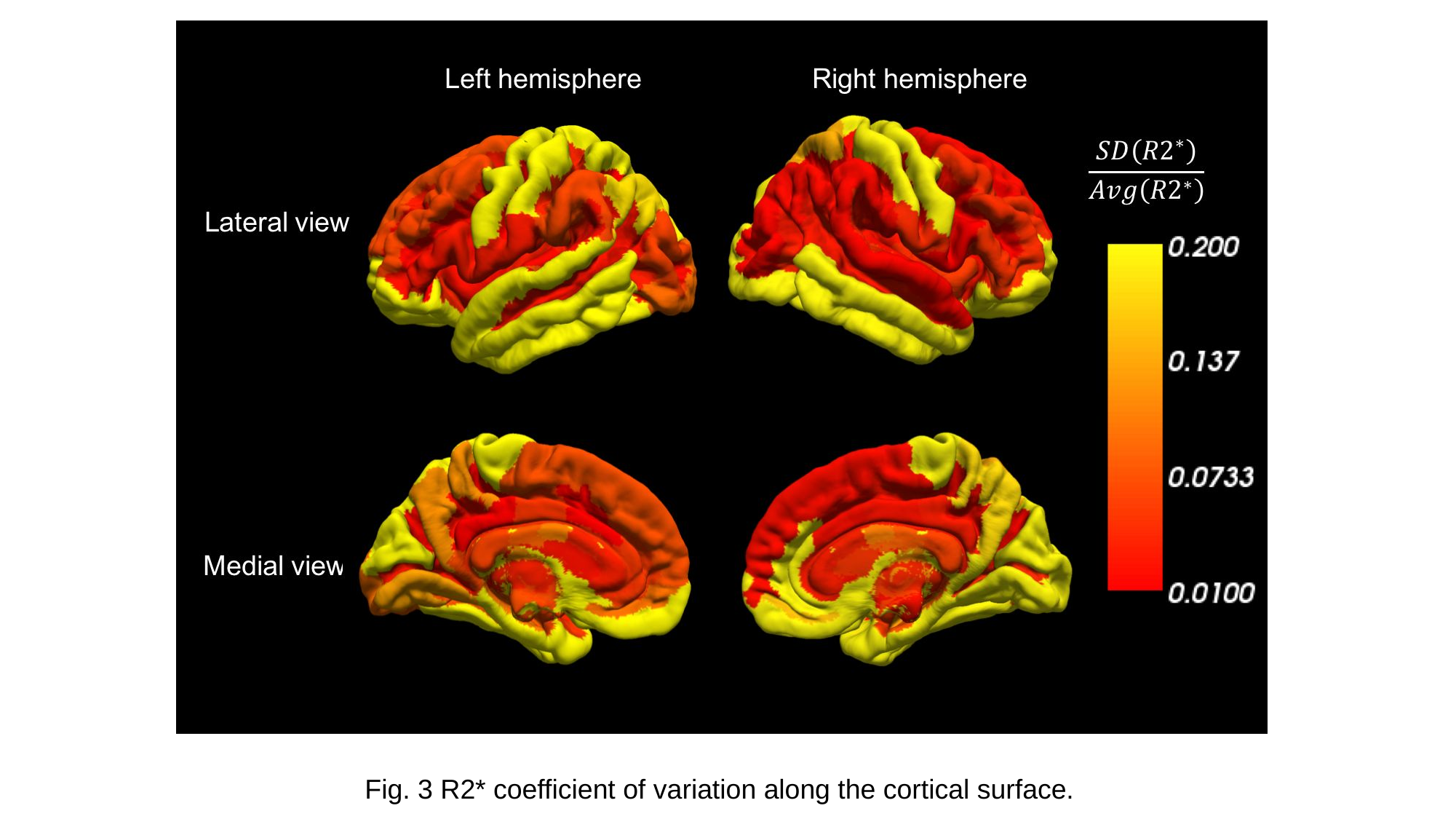

Fig. 3 R2* coefficient of variation along the cortical surface.

## Slide 4
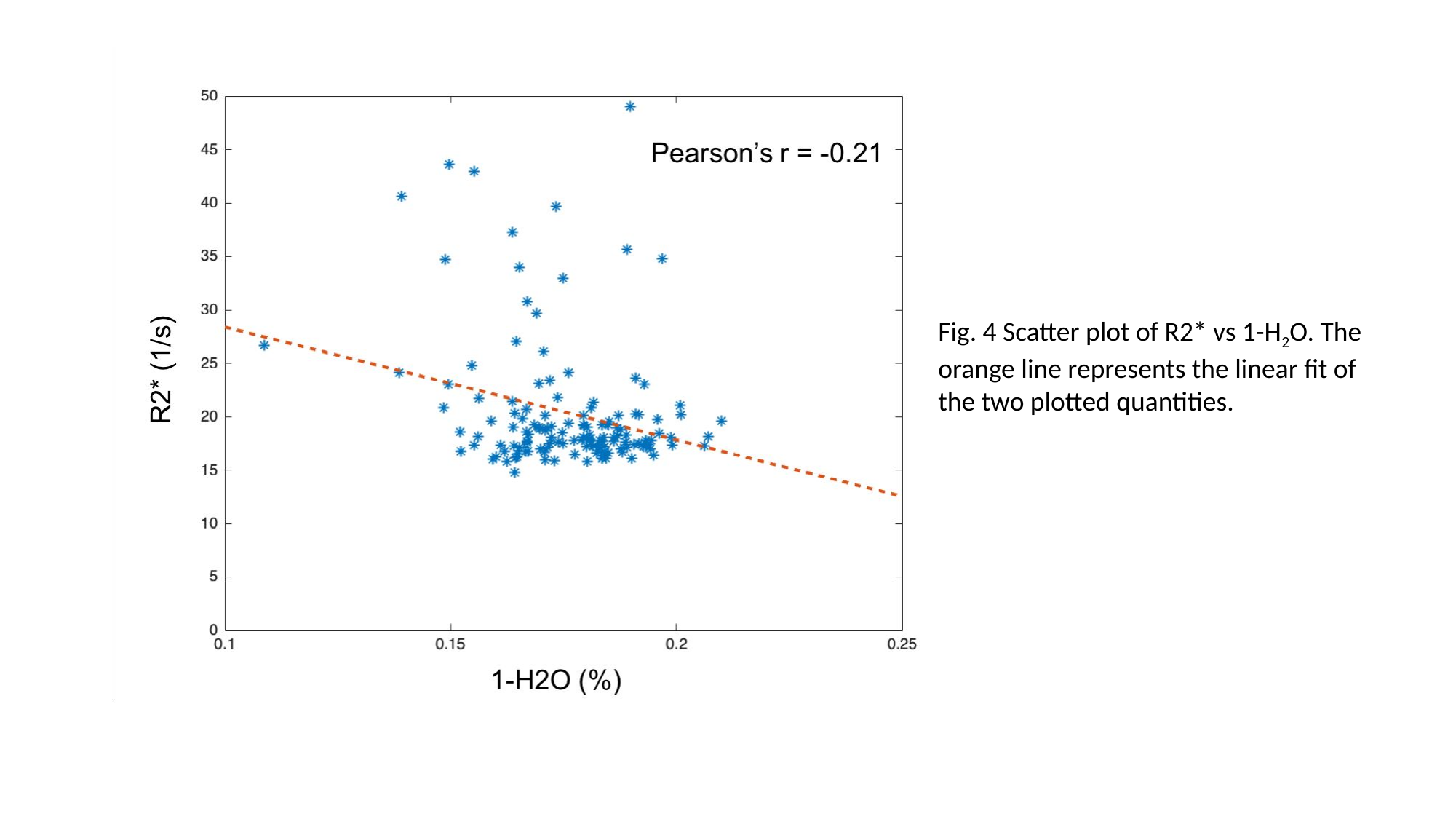

Fig. 4 Scatter plot of R2* vs 1-H2O. The orange line represents the linear fit of the two plotted quantities.
